# Development and Validation of a Risk Scoring System for Cephamycin-Associated Hemorrhagic Events

**DOI:** 10.1038/s41598-019-49340-5

**Published:** 2019-09-09

**Authors:** Tong-Ling Chien, Fei-Yuan Hsiao, Li-Ju Chen, Yu-Wen Wen, Shu-Wen Lin

**Affiliations:** 1 Graduate Institute of Clinical Pharmacy, College of Medicine, National Taiwan University, Taipei, Taiwan; 2 School of Pharmacy, National Taiwan University, Taipei, Taiwan; 3 Department of Pharmacy, National Taiwan University Hospital, Taipei, Taiwan; 4 Health Data Research Center, National Taiwan University, Taipei, Taiwan; 5 Clinical Informatics and Medical Statistics Research Center, Chang Gung University, Tao-Yuan, Taiwan

**Keywords:** Epidemiology, Outcomes research

## Abstract

Cephamycin-associated hemorrhages have been reported since their launch. This research aimed to determine risk factors for cephamycin-associated hemorrhagic events and produce a risk scoring system using National Taiwan University Hospital (NTUH) database. Patients who were older than 20 years old and consecutively used study antibiotics for more than 48 hours (epidode) at NTUH between January 1^st^, 2009 and December 31^st^, 2015 were included. The population was divided into two cohorts for evaluation of risk factors and validation of the scoring system. Multivariate logistic regression was used for the assessment of the adjusted association between factors and the outcome of interest. Results of the multivariate logistic regression were treated as the foundation to develop the risk scoring system. There were 46402 and 22681 episodes identified in 2009–2013 and 2014–2015 cohorts with 356 and 204 hemorrhagic events among respective cohorts. Use of cephamycins was associated with a higher risk for hemorrhagic outcomes (aOR 2.03, 95% CI 1.60–2.58). Other risk factors included chronic hepatic disease, at least 65 years old, prominent bleeding tendency, and bleeding history. A nine-score risk scoring system (AUROC = 0.8035, 95% CI 0.7794–0.8275; Hosmer-Lemeshow goodness-of-fit test *p* = 0.1044) was developed based on the identified risk factors, with higher scores indicating higher risk for bleeding. Use of cephamycins was associated with more hemorrhagic events compared with commonly used penicillins and cephalosporins. The established scoring system, CHABB, may help pharmacists identify high-risk patients and provide recommendations according to the predictive risk, and eventually enhance the overall quality of care.

## Introduction

Cephamycins have proven themselves as potential alternatives to carbapenems in treating infections caused by extended-spectrum beta-lactamase-producing isolates^1,2^. However, increased risk of bleeding with the use of cephamycins has been reported^3–11^. The potential mechanism of this risk may be attributed to that cephamycins may cause hypoprothrombinemia via inhibition of the growth of vitamin K – producing intestinal bacteria or inhibition of vitamin K – epoxide reductase and vitamin K – dependent carboxylase^12^. According to the internal statistics, cephamycins took part in 8% among all intravenous antibiotics during 2009–2013 in National Taiwan University Hospital (NTUH), which implied the significance of cephamycin-related bleeding risk.

In addition, our previous study using Taiwan's National Health Insurance Research Database (NHIRD) found cephamycin was associated with increased risk of hemorrhagic events. Patients on anticoagulants, in poor nutritional status, with underlying liver failure, and encountering previous hemorrhagic events were recognized as possible risk factors. Nevertheless, this study was limited to the lack of laboratory data due to the nature of NHIRD^12^. Also, patients with combination of two or more risk factors are frequently seen in daily practice. The cumulative effects brought by possible risk factors were not further discussed in this study. On top of that, in the era of emphasizing cost-effective medical care, precisely defining and monitoring patients with higher risk become much more important.

Therefore, this study aims to define the risk factors for cephamycin-associated hemorrhage using NTUH electronic medical record (EMR), and to establish a risk scoring system that can be adapted in clinical settings to enhance the safe use of cephamycin.

## Methods

### Data source

Data used in this study was obtained from the NTUH EMR. The database comprises person-level records of demographic information, medication utilizations in NTUH, medical history, laboratory examinations and imaging study results. This retrospective study was approved by Research Ethic Committee of NTUH with the waiver of informed consent. The research team was authorized to have the access to NTUHEMR (REC Number: 201312061RINB). All methods were carried out in accordance with relevant guidelines and regulations.

### Study design and subjects

A retrospective cohort design was applied. Patients aged ≥20 years who continuously used injection forms of the study antibiotics for ≥48 hours in the emergency or inpatient department in NTUH during January 1^st^, 2009 to December 31^st^, 2015 were included. Study antibiotics included cephamycins (cefmetazole, flomoxef, cefoxitin and cefoperazone) and reference antibiotics (amoxicillin/clavulanate, ampicillin/sulbactam, cefuroxime, cefotaxime and ceftriaxone). Reference antibiotics were chosen under the consideration that they should have similar antibacterial spectrum and therapeutic roles with cefmetazole and flomoxef in treating infectious diseases, have no structures known to induce hypoprothrombinemia, and have rare adverse hemorrhagic events reported previously. Data were provided by Department of Pharmacy and Integrated Medical Database at National Taiwan University Hospital (NTUH-iMD).

The first prescription day of study antibiotics was assigned as the index date. The index date plus the following ten days composed the observational period^12,13^. We excluded patients who changed antibiotics from one study antibiotic to another during the observational period. Those who had observational period beyond the study period or prior use of study antibiotics within 30 days of the index date were also excluded. The treatment course was defined as the duration from the index date to the day of discontinuing study antibiotics.

We further divided all identified study subjects into two cohorts based on their index year. The 2009–2013 cohort was used for identifying risk factors and development of the scoring system while the 2014–2015 cohort was used for validation of the scoring system.

### Outcome measurements

Study outcome was the occurrence of any hemorrhagic event during the observational period, which was defined through a two-step process. Firstly, we used the hemorrhagic-related diagnosis codes (International Classification of Diseases, Ninth Revision, Clinical Modification (ICD-9-CM)) documented within the observational period to detect the suspected hemorrhagic events. These events were further examined by chart review to see if the hemorrhage developed after the initiation of study antibiotics.

### Covariates

Covariates including patient demographics, information of index stay, underlying diseases and conditions, concurrent medications/treaments, concurrent antibiotic, and indication of study antibiotics were also collected. Underlying diseases and conditions included chronic hepatic disease, chronic renal diseases, coagulopathy, history of operation, international normalized ratio (INR) prologation, hemorrhagic events, thrombocytopenia, hypoalbuminemia, hepatic dysfunction and renal dysfunction. History of hemorrhagic events was defined by hemorrhagic-related ICD-9-CM codes within 180 days prior to index date or positive stool occult blood test within 7 days prior to index date. Chronic hepatic/renal diseases, and coagulopathy were identified by ICD-9-CM codes coded before index date^14^ and the available laboratory data acquired within 7 days prior to the index date^13,15^.

Concurrent medications/treatments within 7 days prior to index date include antiplatelets, anticoagulants, Vitamin K1, total parenteral nutrition (TPN), proton pump inhibitor (PPI), histamine-2 (H_2_) blockers, tranexamic acid, clotting factors, steroids, nonsteroidal anti-inflammatory drugs (NSAIDs), packed red blood cells (PRBC) and fresh frozen plasma (FFP). We also retrieved any chemotherapy received by the study subjects within 180 days prior to the index date. Use of concurrent antibiotics was defined as any antibiotics other than study antibiotics prescribed for at least 72 hours during treatment course of study antibiotics. Indication of antibiotics was categorized based on infection sites identified through physicians’ orders when prescribing antibiotics and diagnosis codes upon discharge.

### Statistical analysis

Descriptive statistics including Chi-square and Mann-Whitney U tests were estimated in both derivation and validation cohorts. Univariate logistic regression was used to examine the unadjusted association between independent variables and the outcome. The significance level in univariate analyses was set at 0.25. Factors shown to be statistically significant in univariate analyses were selected as candidates for multivariate logistic regression. Stepwise strategy with slentry level at 0.15 and slstay level at 0.05 was applied for model selection. Factors with *p*-value < 0.05 in the adjusted analysis were considered statistically significant and were incorporated into the risk prediction model. The multivariate logistic regression coefficients of each factor were transformed into a risk score by divided the regression coefficients by the smallest regression coefficient among the variables and then rounding the quotients to the nearest integer when developing a risk scoring system.

Model performance consisting of the area under the receivers operating curve (AUROC) and Hosmer-Lemeshow goodness-of-fit test was further assessed. Moreover, the Youden’s index was applied in determining the optimal cutoff value of the risk score to best differentiate the outcomes. Cochran-Armitage trend test was used to evaluate if the score and occurrence of outcome were positively correlated. All data were analyzed using Microsoft Excel 2010 (Microsoft Corp., Redmond, WA, USA) and SAS 9.4 (SAS Institute Inc., Cary, NC, USA).

## Results

### Patient demographics

**Figure 1:**
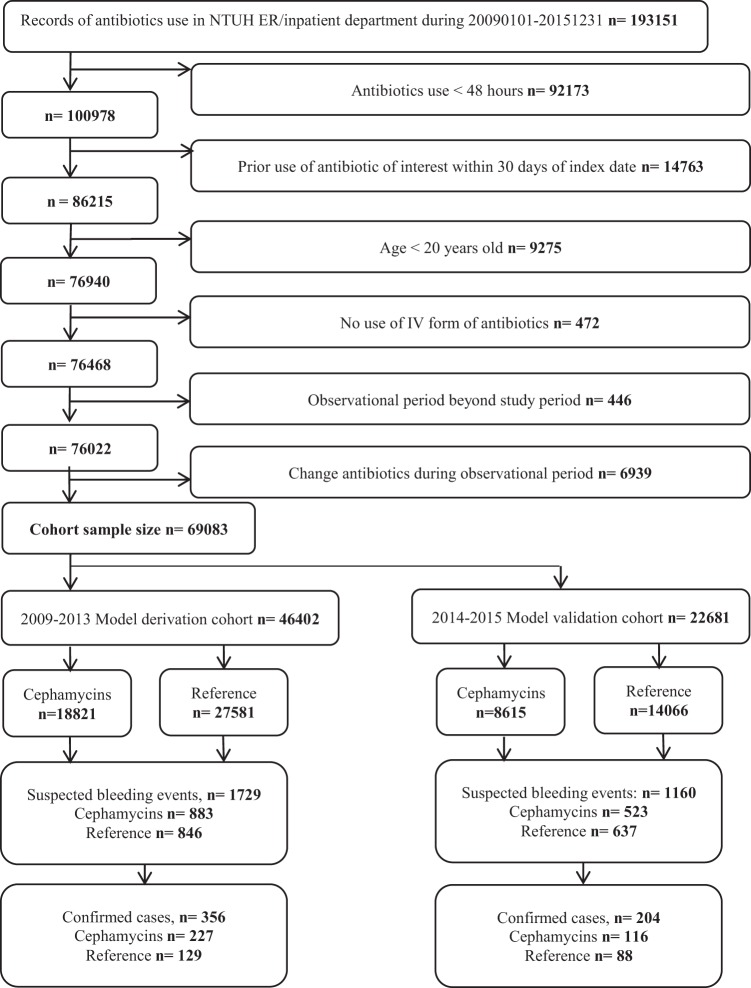
Study flow of cohort enrollment.

The initial cohort included 193151 episodes with exposures to study antibiotics. After applying all the inclusion and exclusion criteria, there were 69083 episodes identified. The cohort was divided into two sub-cohorts as the 2009–2013 derivation cohort (46402 episodes from 37094 individuals) and the 2014–2015 validation cohort (22681 episodes from 19201 individuals) according to the index date. In the derivation cohort, there were 18821 episodes exposed to cephamycins and 27581 episodes exposed to reference antibiotics. Among them, 227 (1.21%) and 129 (0.47%) hemorrhagic events occurred during exposure to cephamycins and reference antibiotics. Gastrointestinal bleeding was the most common type of hemorrhage events (303/356, 85.11%). In the validation cohort, there were 8615 episodes exposed to cephamycins and 14066 episodes exposed to reference antibiotics. Among them, 116 (1.35%) and 88 (0.63%)  bleeding occurred during exposure to cephamycins and reference antibiotics (Fig. 1).

Compared to users of reference antibiotics, cephamycins users were younger (median age 61.5 years in cephamycins vs. 64.7 years in reference antibiotics, *p* < 0.0001) and more likely to have chronic hepatic diseases (7.91% vs. 4.74%, *p* < 0.0001). In terms of concurrent medications, cephamycins users were less likely to concurrently receive antiplatelets (4.08% vs. 10.84%, *p* < 0.0001) and anticoagulants (2.27% vs. 5.73%, *p* < 0.0001). On the contrary, cephamycins users were prone to concurrently receive vitamin K_1_ (1.84% vs. 1.16%, *p* < 0.0001), TPN (10.48% vs. 2.11%, *p* < 0.0001), PPIs (22.15% vs. 9.14%, *p* < 0.0001), H_2_-blockers (8.53% vs. 5.67%, *p* < 0.0001) and tranexamic acid (6.34% vs. 5.76%, *p* = 0.009) (Table 1).

**Table 1:** Patient characteristics of study antibiotics users.

	Derivation		Validation	
	Cephamycins	Reference	Cephamycins	Reference
	n = 18821 (%)	n = 27581 (%)	n = 8615 (%)	n = 14066 (%)
**Demographics**				
Age (year)^a^	61.5 (49.7–73.8)^**^	64.7 (50.9–78.1)	63.2 (52.1–74.9)^**^	64.8 (52.3–78.2)
≥65 y/o	8040 (42.72)^**^	13634 (49.43)	3882 (45.06)^**^	6965 (49.52)
<65 y/o	10781 (57.28)^**^	13947 (50.57)	4733 (54.94)^**^	7101 (50.48)
Gender, males	9495 (50.45)^**^	15987 (57.96)	4491 (52.13)^**^	7995 (56.84)
Height (cm)^a^	161.0 (155.0–167.0)^**^	161.0 (155.8–168.0)	161.0 (155.0–167.7)^**^	161.6 (155.2–168.0)
Weight (kg)^a^	59.1 (51.8–67.6)	59.1 (51.8–67.6)	59.8 (52.2–68.6)	59.9 (52.0–68.6)
BMI (kg/m^2^)^a^	22.8 (20.7–25.4)^**^	22.8 (20.4–25.2)	23.0 (20.7–25.6)^*^	22.9 (20.4–25.7)
**Index stay**				
Length of stay (day)^a^	11 (7–19)^**^	13 (8–23)	5 (3–8)^**^	5 (3–8)
Antibiotics treatment course (day)^a^	5 (3–8)	5 (3–7)	5 (3–8)^**^	4 (3–7)
**Underlying diseases and conditions**				
Chronic hepatic disease	1488 (7.91)^**^	1307 (4.74)	874 (10.15)^**^	895 (6.36)
Chronic renal disease	793 (4.21)^**^	1661 (6.02)	428 (4.97)^**^	1071 (7.61)
Coagulopathy	7 (0.04)	17 (0.06)	2 (0.02)	13 (0.09)
Operation history^b^	2262 (12.02)^**^	2825 (10.24)	1009 (11.71)^**^	1408 (10.01)
INR prolongation^c^	243 (1.29)^*^	294 (1.07)	199 (2.31)	351 (2.5)
Hemorrhagic events^d^	2081 (11.06)^**^	2326 (8.43)	1265 (14.68)^**^	1585 (11.27)
Thrombocytopenia	642 (3.41)	1021 (3.70)	498 (5.78)	830 (5.9)
Hypoalbuminemia	336 (1.79)^**^	399 (1.45)	442 (5.13)	673 (4.78)
Hepatic dysfunction	1032 (5.48)^**^	604 (2.19)	1212 (14.07)^**^	798 (5.67)
Renal dysfunction	654 (3.47)^**^	1430 (5.18)	802 (9.31) ^**^	1881 (13.37)
**Concurrent medications/treaments (within 7 days prior to index date)**				
Antiplatelets	768 (4.08)^**^	2991 (10.84)	295 (3.42)^**^	1300 (9.24)
Anticoagulants	427 (2.27)^**^	1581 (5.73)	258 (2.99)^**^	772 (5.49)
Vitamin K_1_	346 (1.84)^**^	320 (1.16)	99 (1.15)	137 (0.97)
Total parenteral nutrition (TPN)	1973 (10.48)^**^	582 (2.11)	675 (7.84)^**^	232 (1.65)
Proton pump inhibitors (PPI)	4169 (22.15)^**^	2522 (9.14)	2764 (32.08)^**^	1790 (12.73)
Histamine-2 (H_2_) blockers	1605 (8.53)^**^	1564 (5.67)	199 (2.31)^**^	486 (3.46)
Tranexamic acid	1194 (6.34)^**^	1588 (5.76)	642 (7.45) ^*^	933 (6.63)
Clotting factors	13 (0.07)	19 (0.07)	4 (0.05)	13 (0.09)
Steroids	1484 (7.88)^**^	4357 (15.80)	696 (8.08)^**^	2357 (16.76)
Nonsteroidal anti-inflammatory drugs (NSAIDs)	3274 (17.40)^**^	6357 (23.05)	1137 (13.2)^**^	3052 (21.7)
Packed red blood cells (PRBC)	35 (0.19)	55 (0.20)	160 (1.86)	235 (1.67)
Fresh frozen plasma (FFP)	14 (0.07)	15 (0.05)	65 (0.75)	78 (0.55)
Chemotherapy^d^	3070 (16.31)	4529 (16.42)	1645 (19.09)	2629 (18.69)
**Concurrent antibiotics (≥3 days in treatment course)**				
Metronidazole	685 (3.64)^**^	1194 (4.33)	135 (1.57)^**^	617 (4.39)
Aminoglycosides	475 (2.52)^**^	319 (1.16)	315 (3.66)^**^	66 (0.47)
Glycopeptides	122 (0.65)^**^	312 (1.13)	94 (1.09)	135 (0.96)
Macrolides	81 (0.43)^**^	172 (0.62)	58 (0.67)^**^	28 (0.2)
Sulfonamides	92 (0.49)^**^	380 (1.38)	33 (0.38)^**^	177 (1.26)
**Indications of study antibiotics**				
Genitourinary	3768 (20.02)^**^	3948 (14.31)	1149 (13.34)^**^	2144 (15.24)
Gastrointestinal and Intra-abdominal	9922 (52.72)^**^	4567 (16.56)	2193 (25.46)^**^	1105 (7.86)
Respiratory tract	1444 (7.67)^**^	10355 (37.54)	535 (6.21)^**^	4350 (30.93)
Central nervous system	116 (0.62)^**^	405 (1.47)	12 (0.14)^**^	77 (0.55)
Circulatory	663 (3.52)^**^	1615 (5.86)	88 (1.02)^**^	220 (1.56)
Skin and soft tissue	411 (2.18)^**^	3322 (12.04)	173 (2.01)^**^	1678 (11.93)
Bone and joint	240 (1.28)^**^	617 (2.24)	9 (0.1)^**^	58 (0.41)
Prophylaxis	1882 (10.00)	2990 (10.84)	1701 (19.74)^**^	815 (5.79)
Empirical	3851 (20.46)^**^	6018 (21.82)	3936 (45.69)^**^	5679 (40.37)

^a^Median (IQR); ^b^Within previous 30 days; ^c^Within previous 7 days; ^d^Within previous 180 days; ^*^p-value < 0.05; ^**^p-value < 0.01 compared with reference antibiotics.

### Derivation of risk prediction model for cephamyins-associated hemorrhage

The final risk prediction model with AUROC of 0.8072 contained five variables that were all statistically significantly associated with hemorrhage (*p* < 0.0001) in the multivariate logistic regression analyses. The regression coefficients of all variables in the risk prediction model were transformed into integer risk scores demonstrated in Table 2. Patients using cephamycins, aged 65 years or above, afflicted with chronic hepatic disease, having history of bleeding within 180 days prior to the index date, and showing bleeding tendency, which was indicated by prescriptions of PPIs, tranexamic acid or clotting factors, within 7 days prior to the index date had an increased risk score by one, two, or four points. A risk scoring system named CHABB (stands for use of Cephamycins, underlying chronic Hepatic diseases, Aged 65 years or above, Bleeding tendency, and Bleeding history in the preceding 180 days of the index date) with 5 items was established and the sum risk scores ranged from 0 to 9.

**Table 2:** Development of scoring system for bleeding risk using multivariate logistic regression model.

Predictor Variable	Adjusted OR (95% CI)	*p*-value	Regression Coefficient	Risk Score
**Study antibiotics**				
Reference^¶^	1	—	0	0
Cephamycins^§^	2.03 (1.60–2.58)	<0.0001	0.3542	1
**Chronic hepatic disease**				
No	1	—	0	0
Yes	2.08 (1.54–2.81)	<0.0001	0.3661	1
**Age (years)**				
<65	1	—	0	0
≥65	1.66 (1.31–2.09)	<0.0001	0.253	1
Bleeding tendency*				
No	1	—	0	0
Yes	2.46 (1.94–3.12)	<0.0001	0.4506	2
**History of bleeding** ^**¥**^				
No	1	—	0	0
Yes	6.84 (5.38–8.68)	<0.0001	0.9612	4
**Model performance**				**Total Score**
AUROC	0.8072 (95% CI, 0.7816–0.8329)			0~9
Hosmer-Lemeshow test	0.1044			

^¶^Amoxicillin/clavulanic acid, ampicillin/sulbactam, cefuroxime, cefotaxime, ceftriaxone; ^§^Cefmetazole, flomoxef; ^¥^During previous 180 days; *Indicated by prescriptions of proton pump inhibitors/tranexamic acid/clotting factors during prior 7 days.

### Validation of risk prediction model for cephamyins-associated hemorrhage

**Figure 2:**
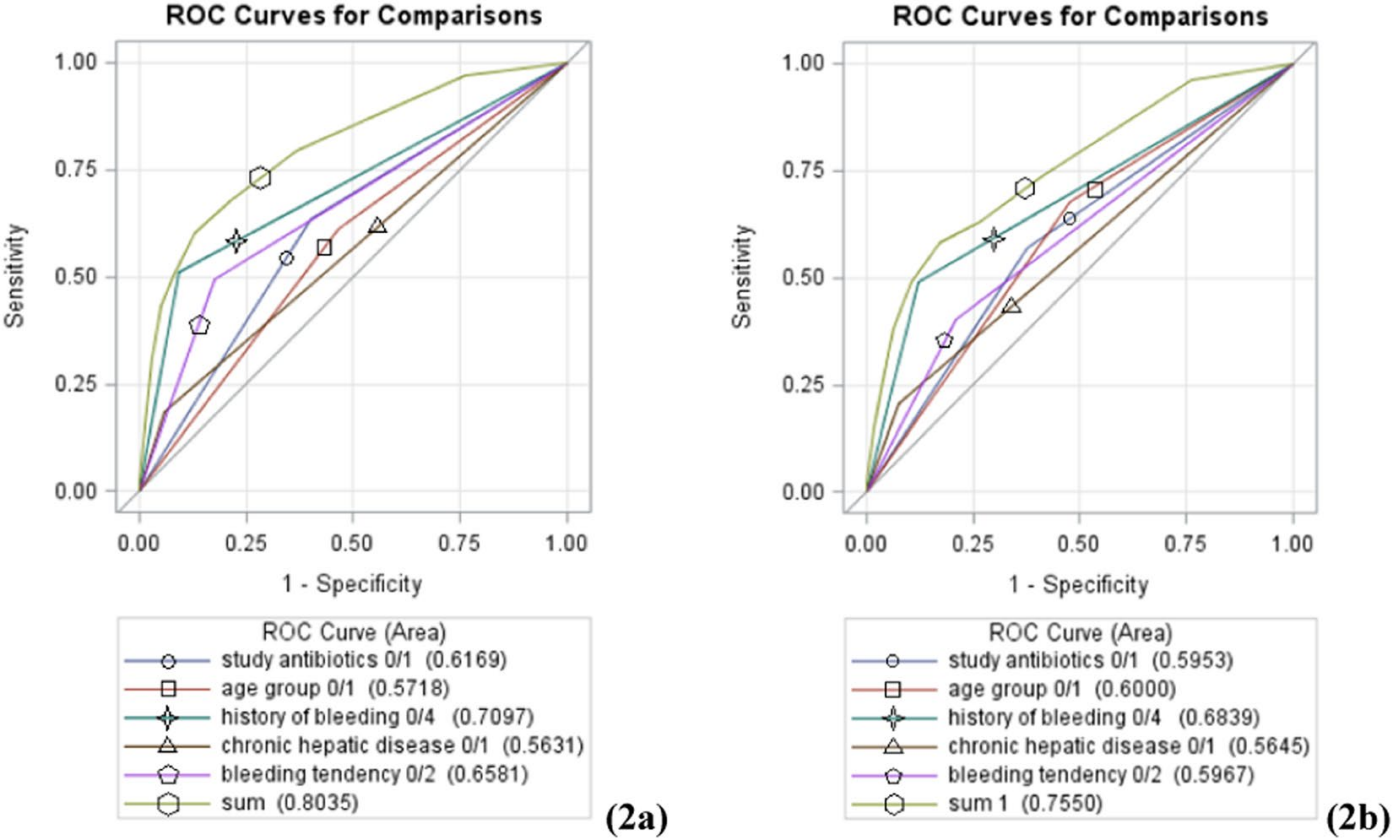
Receivers operating characteristics (ROC) curves of risk score in derivation cohort (**a**) validation cohort (**b**).

**Figure 3:**
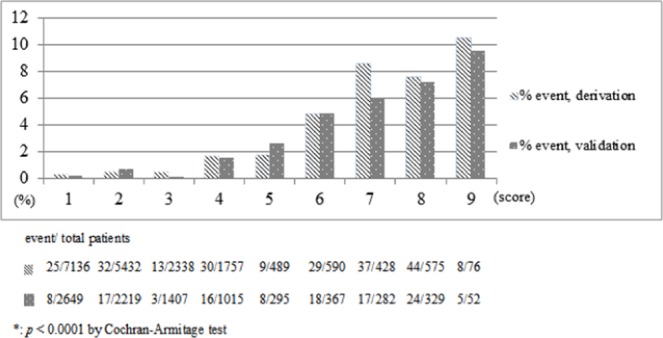
Increasing risk of bleeding events with increasing risk score among cephamycin users.

In the evaluation of predictive accuracy of the risk prediction model for cephamycins-associated hemorrhage, CHABB was able to achieve an AUROC of 0.8035 and 0.7550 in the derivation and validation cohorts, respectively, revealing that the sum risk scores lent satisfactory to great validity to the prediction of cephamycins-associated hemorrhage risk. The predictions made from the risk model were in alignment with the observed outcomes suggested by Hosmer-Lemeshow tests (Table 3) and turned out to be the most accurate when all five items were all included (Fig. 2). The cutoff score suggested by Youden’s index was 6 points among cephamycins users across both cohorts. As depicted in Fig. 3, the counts of hemorrhagic events notably went up as the risk score increased in both cohorts. It indicated that the risk to developing hemorrhagic events was positively correlated to the risk score, which was statistically significant proved by Cochran-Armitage trend tests (*p* < 0.0001) as well. Moreover, we further divided patients in both cohorts into three risk groups, including low (with risk scores from zero to three), medium (with risk scores from four to five) and high risk (with risk scores equal to six or above) groups, using the event percentages of 1% and 4% as cut-offs. And the results revealing that the higher the score, the greater the possibility of developing hemorrhagic events had parallel in those who have not been categorized into groups.

**Table 3:** Validation of the risk scoring system.

Predictor Variable	Risk Score	AUROC (95% CI)	
		Derivation	Validation
**Study antibiotics**			
Reference^¶^	0	0.6169 (0.5918–0.6420)	0.5953 (0.5610–0.6295)
Cephamycins^§^	1		
**Chronic hepatic disease**			
No	0	0.5631 (0.5428–0.5833)	0.5645 (0.5367–0.5924)
Yes	1		
**Age (years)**			
<65	0	0.5718 (0.5463–0.5973)	0.6000 (0.5677–0.6324)
≥65	1		
Bleeding tendency*			
No	0	0.6581 (0.6321–0.6842)	0.5967 (0.5628–0.6305)
Yes	2		
**History of bleeding** ^**¥**^			
No	0	0.7097 (0.6837–0.7358)	0.6839 (0.6495–0.7184)
Yes	4		
Total score	0~9	0.8035 (0.7794–0.8275)	0.7550 (0.7198–0.7902)
Hosmer-Lemeshow test		*p* = 0.1044	*p* = 0.0641

^¶^Amoxicillin/clavulanic acid, ampicillin/sulbactam, cefuroxime, cefotaxime, ceftriaxone; ^§^Cefmetazole, flomoxef; ^¥^During previous 180 days; *Indicated by prescriptions of proton pump inhibitors/tranexamic acid/clotting factors during prior 7 days.

## Discussion

This is a longitudinal study attempting to identify risk factors for cephamycin-associated hemorrhagic events in a teaching hospital in Taipei. The identified risk factors further served as the foundation for the development of a risk scoring system connecting the use of cephamycins and the possible related hemorrhages. To the best of our knowledge, this is the first study with the largest population in evaluation of the risk factors for cephamycin-associated hemorrhagic events and in development and validation of the nine-score risk score system.

We have identified risk factors for cephamycin-associated hemorrhagic events and established a nine-score risk scoring system named CHABB. Our findings were similar to our previous study conducted by Chen *et al*. in 2014 using Taiwan’s NHIRD^12^. In Chen’s study, use of anticoagulants or history of bleeding events in preceding 180 days of the index date, malnourished status and hepatic failure were suggested as risk factors. Among those factors, nutrition status was determined using the surrogate indicator of TPN due to the nature of NHIRD that laboratory data was not available. In our study, nutrition status was determined by serum albumin level, and a normal value was assigned for patients who were lack of records, as was done in Strom *et al*.'s study^16^. However, hypoalbuminemia was not retained in the final multivariable model in our case. Use of anticoagulants and antiplatelet agents were included in stepwise selection for their clinical importance to the outcome, but they were not retained in the final model. This could be physicians’ preference not prescribing cephamycins in patients under long-term anticoagulants or antiplatelet agents to avoid bleeding problems. Also, there may be a healthy-worker bias that patients under anticoagulants or antiplatelet agents persistently may be less likely to develop bleeding diatheses.

Some of the factors shown to be related to hemorrhages were interesting but not surprising. We expected anti-secretion agents of PPIs and agents affecting hemostasis such as clotting factors and tranexamic acid to be protective factors. Nevertheless, these factors were risk factors in our study. Similar results were observed in Brown *et al*. that the use of antiulcer therapy was associated with the highest odds ratio^10^. Considering PPIs, clotting factors and tranexamic acid essentially stabilized the hemostatic process but trended toward higher odds ratios in our regression, we assumed the underlying conditions resulted in the use of these agents were similar. Therefore, we merged the use of either kind of these medications into one variable named bleeding tendency. Judging from the AUROC, the new variable possessed similar ability in explaining the outcome of interest, meanwhile confirming our assumption to some extent.

We chose the scoring system as the presentation format because of its straightforwardness to be used in clinical setting. The indices we adapted to assess the model performance were widely used in building a scoring system^17–24^. Our study has large sample size with relative low incidence of the outcome. Although the calibration result in the 2014–2015 cohort had only a marginal *p*-value indicating the scoring system fit well, we need to understand that the test was based on the foundation of hypothesis testing. That is, the possibility that we make type II error may not be low and results of the statistical evaluation can be sensitive to the differences between the observed and predicted values. After all, the results in both cohorts suggested an appropriate fit of the scoring system.

Despite numerous methodological merits of our study, there were some limitations needed to be mentioned. First, due to its retrospective design, information bias might have occurred in outcome ascertainment. Clinicians may have handled minor hemorrhages before they progressed into serious events. Clinicians may not mention minor events in the discharge notes as well. On the other hand, certain hemorrhagic diagnoses might be part of medical history from previous visits, so they were actually not documented in medical chart. These patients were selected by hemorrhagic-related codes but were then excluded during chart review. Second, we might have included patients who had been prescribed with one of the study antibiotics at medical facilities other than NTUH during the screening period. Third, external validation by a future prospective study would be warranted as it would make this scoring system more relevant for clinical scenario.

In summary, we have established and validated a nine-score risk scoring system to identify potential high risk patients who may develop hemorrhagic events when taking cephamycin. For patients being classified in the high-risk group, we recommend close monitoring of INR and hemorrhagic signs to prevent subsequent catastrophic hemorrhages. In hopes of enhancing the patient care quality, we expect this risk scoring system to be adapted and fully exerted in clinical setting. Future refinement of the scoring system may be considered after the scoring system being prospectively applied clinically for some period of time.
